# Additively Manufactured Scaffolds with Optimized Thickness Based on Triply Periodic Minimal Surface

**DOI:** 10.3390/ma15207084

**Published:** 2022-10-12

**Authors:** Junjie Zhu, Sijia Zou, Yanru Mu, Junhua Wang, Yuan Jin

**Affiliations:** 1 School of Mechatronics Engineering, Henan University of Science and Technology, Luoyang 471003, China; 2 Smart Materials and Advanced Structure Laboratory, School of Mechanical Engineering and Mechanics, Ningbo University, Ningbo 315211, China

**Keywords:** triply periodic minimal surface, porous scaffolds, optimized thickness, mechanical properties, permeability

## Abstract

Triply periodic minimal surfaces (TPMS) became an effective method to design porous scaffolds in recent years due to their superior mechanical and other engineering properties. Since the advent of additive manufacturing (AM), different TPMS-based scaffolds are designed and fabricated for a wide range of applications. In this study, Schwarz Primitive triply periodic minimal surface (P-TPMS) is adopted to design a novel porous scaffold according to the distribution of the scaffold stress under a fixed load with optimized thickness to tune both the mechanical and biological properties. The designed scaffolds are then additively manufactured through selective laser melting (SLM). The micro-features of the scaffolds are studied through scanning electron microscopy (SEM) and micro-computed tomography (CT) images, and the results confirm that morphological features of printed samples are identical to the designed ones. Afterwards, the quasi-static uniaxial compression tests are carried out to observe the stress–strain curves and the deformation behavior. The results indicate that the mechanical properties of the porous scaffolds with optimized thickness were significantly improved. Since the mass transport capability is important for the transport of nutrients within the bone scaffolds, computational fluid dynamics (CFD) are used to calculate the permeability under laminar flow conditions. The results reveal that the scaffolds with optimized structures possess lower permeability due to the rougher inner surface. In summary, the proposed method is effective to tailor both the mechanical properties and permeability, and thus offers a means for the selection and design of porous scaffolds in biomedical fields.

## 1. Introduction

Various types of porous structures can be found in natural biomaterials, such as bones, wood, and honeycombs [1]. In recent years, porous structures began attracting increasing attention in many industrial fields thanks to their high stiffness-to-weight ratio, excellent load bearing capacity, and high energy absorption [2]. Benefiting from these advantages, many studies focused on the application of porous structures in bone scaffolds [3,4]. The increasingly growing traumatic injuries and congenital defects increased the need for bone-mimicking scaffolds [5,6]. Therefore, the investigation on the design and manufacture of porous bone scaffolds is necessary and promising.

Porous scaffolds can be classified based on the regularity of their internal architectures, including the stochastic scaffolds and the non-stochastic scaffolds [7]. The stochastic scaffolds involve the pores with random shapes and size distributions, such as those formed using salt-leaching and gas-foaming techniques [8]. However, stochastic scaffolds possess some disadvantages, such as uncontrollable mechanical properties and local stress concentration. By contrast, the non-stochastic scaffolds are defined as complex architectures containing an array of element bodies that are repeatedly aligned in the 3D space, such as cubic, octahedron, and truncated [9]. The physical properties of non-stochastic structures largely depend on the design of internal characteristics, including pore size, pore shape, and relative density [10]. Therefore, the properties of non-stochastic scaffolds can be effectively tuned by rational design of the pore shape, relative density, and specific surface area, and thus, these scaffolds are universally adopted in the design of bone scaffolds.

There are many factors to consider when designing bone scaffolds, such as geometry, mechanical properties and permeability [11]. Meanwhile, it is also necessary to consider the manufacturing process of the bone scaffolds to achieve desirable accuracy. In recent years, additive manufacturing (AM) provided a feasible method for producing complex parts with high precision and efficiency [12]. The metallic powders are fused by the laser beam and assembled to form three-dimensional metal parts with rough surfaces that are adapted to the growth needs of cells. Selective laser melting (SLM) is the most frequently used AM technique for the fabrication of metal implants [13]. Numerous studies show that SLM offers a number of advantages, including high mechanical load capacity, tunable density, excellent precision, low cost, ready-to-use product, the ability to tune properties by adjusting process parameters, and the ability to produce complex structures with minimal support structures [14]. Bobbert et al. [15] designed several different types of porous scaffolds, which were then manufactured by SLM to mimic the topological, mechanical, and mass transport properties of trabecular bone to a great degree. The result showed that SLM-printed scaffold had high printing accuracy and could meet the mechanical properties of bone scaffolds.

In recent years, triply periodic minimal surfaces (TPMS) drew tremendous attention to the design of bone scaffolds due to their capabilities in the control of pore size and porosity [16]. There are many types of TPMS structures, such as primitive, gyroid, diamond, and IWP [17]. These TPMS structures have smooth surfaces, uniform curvatures, and self-supported features that are very suitable for being manufactured by AM. In addition, the mechanical properties, fatigue resistance, and permeability of TPMS are also excellent [18]. Moreover, TPMS structures can provide large surface area and pore interconnectivity, which are conducive to cell adsorption and growth [19]. Wang et al. [20] designed several groups of primitive-based scaffolds that were then fabricated by SLM. The results show that the mechanical properties and permeability of the scaffolds were good to be used as bone tissue replacement. Al-Ketan et al. [21] analyzed the mechanical performance of TPMS-based structures and strut-based structures, and they pointed out that the TPMS-based structures possessed superior mechanical properties.

Bone scaffolds with proper mechanical properties should have adequate load-bearing capacity and fatigue resistance, as well as the ability to reduce stress shielding. Various mechanical properties of TPMS-based scaffolds were extensively investigated. Al-Ketan et al. [21] designed several periodic scaffolds with different classes of topologies and investigated the mechanical properties of these periodic cellular structures by quasi-static compression experiments, and the results indicate that the sheet-based structures had superior mechanical properties among all the tested structures. Sun et al. [22] investigated the mechanical properties and deformation mechanisms of three types of TPMS sheet-based structures and found that the compressive mechanical properties had an approximately positive relationship with the relative density. In addition, Novak et al. [23] characterized the stress–strain relationship and deformation behavior of TPMS-based sheet structures under both quasi-static and dynamic loading. They found that different types of specimens had the same global deformation behaviors in both testing. Li et al. [24] designed some Gyroid-based structures and investigated the compressive behavior of the printed specimens experimentally under static and dynamic loading. They concluded that the mechanical properties, including yield stress, plateau stress, and energy absorption of the printed structures, exhibit certain loading rate sensitivity. Overall, the mechanical properties of TPMS-based scaffolds are significantly affected by their topologies, so it is an effective method to enhance the mechanical performance by adjusting the cell topology.

Mass transport plays a crucial role in the effective exchange of nutrients and wastes that are required for cell proliferation and differentiation [25]. The permeability of scaffolds is an important factor affecting their ability of nutrient transmission. Therefore, the permeability of the scaffolds needs to be investigated. Santos et al. analyzed the permeability of 3D-printed TPMS scaffolds experimentally and revealed that TPMS structures could achieve higher permeability values compared with other porous structures [26]. Castro et al. [27] considered the permeability as a function of the geometry of TPMS with a combination of numerical and experimental methods. The outcomes revealed that the design of the unit cell had a noticeable effect on permeability. Compared to experimental analyses, CFD recently gained significant attention with the capacity to provide immediate results as an alternative and economical approach. Numerous CFD analyses were conducted to evaluate the permeability of various types of scaffolds [28]. Ali et al. [29] investigated the fluid flow within the scaffolds using computational fluid dynamic (CFD) analysis, and the results show that the permeability of the scaffolds was governed by their architecture and any changes in the size of channels would reduce permeability. Asbai-Ghoudan et al. [30] used CFD to calculate permeability for three TPMS-based scaffolds and the results show that permeability increased with porosity at different rates, highlighting the importance of pore distribution and architecture. Ma et al. [14] indicated that the roughness of the scaffolds’ surfaces can influence transmission performance, especially for the scaffolds with small pore sizes.

Despite intensive research on TPMS-based scaffolds, few studies focused on the optimization of the unit cell of TPMS to improve their performance. In recent years, the research on the optimization of the unit cell of TPMS to improve their performance drew great interest. Jia et al. [31] proposed an enhanced design method based on local shell thickening of the unit cell of TPMS. Liu et al. [32] proposed a novel design method for a composite porous structure based on TPMS and geodesic B-spline hollowing. Wang et al. [33] used the Sigmoid function surface splicing method to splice different TPMS structures, and designed three novel types of composite TPMS-based porous structure. The experimental and FEA result indicates that these methods were effective and reliable to obtain the unit cell of TPMS with better mechanical properties. In this paper, a new type of TPMS-based scaffolds is proposed by optimizing the unit cell of Schwarz Primitive TPMS (P-TPMS). The whole scaffold is constructed by arraying the optimized unit cells and then fabricated by SLM. In addition to the mechanical properties, the permeability is also investigated to verify the feasibility of the optimized scaffolds for mimicking the physical properties of human bones. The remainder of the paper is organized as follows: Section 2 introduces the design of the proposed scaffolds and experimental details. Both the experimentally and numerically obtained results are provided and discussed in Section 3. We conclude the paper briefly in Section 4.

## 2. Methodology

### 2.1. Design of P-TPMS Scaffolds

**Figure 1:**
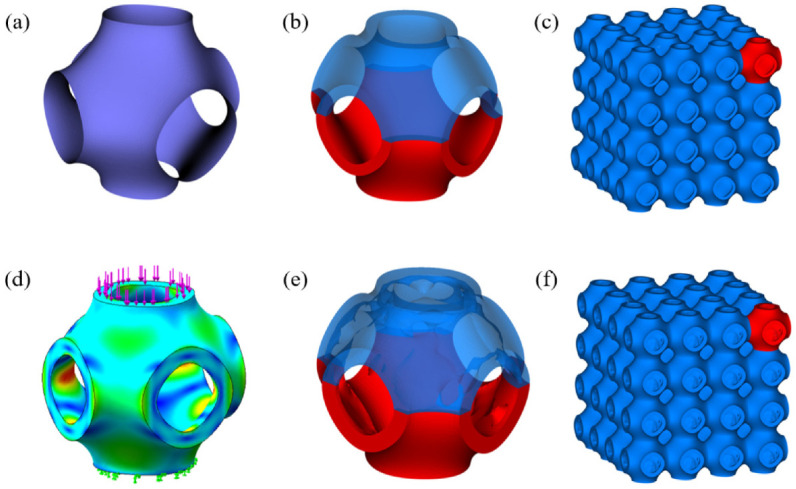
Design of the optimized P-TPMS scaffolds. (**a**) Schwarz Primitive surface; (**b**) P-TPMS unit cell; (**c**) P-TPMS scaffolds with uniform sheet thickness; (**d**) optimized process; (**e**) optimized P-TPMS unit cell; (**f**) P-TPMS scaffolds with optimized thickness.

The P-TPMS was generated in this work according to the mathematical definition as expressed by Equation (1) and as shown in Figure 1a.

(1) $$cos(\frac{2\pi }{L}x)+cos(\frac{2\pi }{L}y)+cos(\frac{2\pi }{L}z)=C$$

where *L* is used to define the unit length, and *C* controls the geometry of the surface, varying from −1.0 to 1.0. Two types of P-TPMS unit cells with a shell thickness of 0.4 mm and 0.6 mm and unit length of 2π mm were initially designed. Subsequently, the designed unit cells were optimized using static stress for simulation by setting a fixed load on the top surface of the models and fixed on the bottom surface, as illustrated in Figure 1d, and then used the topological simulation to optimize the inner surface under relatively small stress in the Solidworks software (Dassault Systems, Waltham, MA, USA). Three different topologically optimized unit cells were obtained by setting different optimization objectives, as shown in Figure 1e. Specifically, the models with 0.4 mm sheet thickness were optimized to 11.5%, 16.4%, and 21.4%, respectively, while the models with 0.6 mm sheet thickness were optimized to 12.6%, 18.2%, and 25.6%, respectively. In order to verify the performance of optimized models, models with the same porosities but uniform sheet thickness were also designed, as shown in Figure 1b. The whole of the scaffolds were designed by arraying the designed unit cells in three directions, as illustrated in Figure 1c,f. To distinguish different scaffolds, they are named according to their sheet thickness and optimization degree. P0.4 and P0.6 represent scaffolds with sheet thickness of 0.4 mm and 0.6 mm, respectively. In addition, A, B, and C represent scaffolds from a low optimization degree to a high optimization degree, while 1 represents the optimized model, and 2 represents the non-optimized model with the same solid volume. For example, the model with shell thickness of 0.4 mm and optimization degree of 11.5% is named as P0.4A1.

### 2.2. Additive Manufacturing of Scaffolds

**Figure 2:**
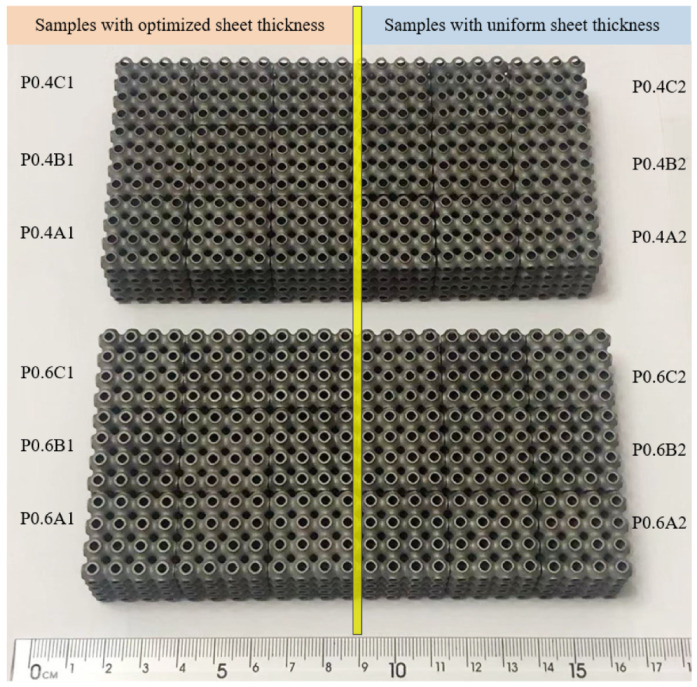
Fabricated samples by SLM.

Figure 2 shows the obtained P-TPMS scaffolds that were manufactured via SLM. All samples were manufactured using a DiMetal-280 machine (LaserAdd Company, Guangzhou, China). Each type of structure was produced three times for the compression testing. The used material was 304L powder, the chemical composition of which is illustrated in Table 1. The powders have nearly spherical shapes with a wide range of particle size distribution, the D10, D50, and D90 of the powder are at 21.42 μm, 33.59 μm, and 51.53 μm, respectively. The process parameters were a laser power of 170 W, scanning speed of 1000 mm/s, layer thickness of 0.03 mm, and beam diameter of 80 μm.

### 2.3. Morphological Characterization

The mass of each printed sample was measured by an electric balance (0.1 g). The volume of each sample could be achieved through dividing mass by the theoretical bulk density (7.8 g/cm^3^). The morphology of the sample was detected by a mirco-computed tomography (CT) scan and scanning electron microscopy (SEM). To reduce the time and cost, only a sample of P0.6B1 was selected as the representative. A micro-CT scanner (Phoenix v|tome|x, General Electric, Frankfurt, Germany) with a 21.36 μm resolution was used to scan the samples with an accelerating voltage of 180 kV, and a current of 200 mA. Additionally, 2D slice images were obtained from the scanning process and then used to analyse the residual powders and inner strcutures.The 3D model was rebuilt to analyse the surface appearance and the manufacturing deviation. SEM (SU5000, Hitachi, Tokyo, Japan) at a 15 KV accelerating voltage was used to observe the surface characteristics of the samples, and images can show the contour and hatching tracks, as well as the powders adhering to the surface.

### 2.4. Quasi-Static Compression Testing

**Figure 3:**
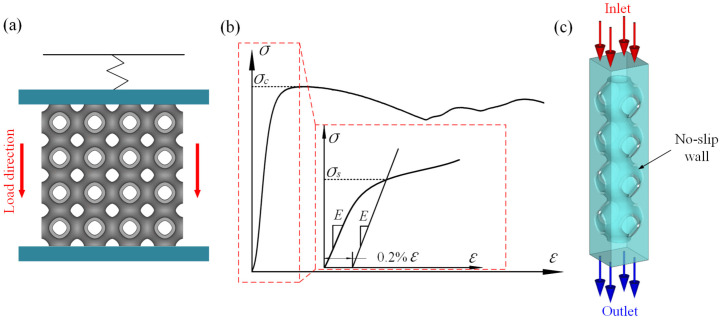
(**a**) Schematic diagram of compressive test; (**b**) schematic of elastic modulus and strength; and (**c**) boundary conditions for CFD analysis.

The samples were compressed at a prescribed loading rate of 2 mm/min using an electronic testing machine (MTS Landmark, Eden Prairie, MN, USA, 50 KN) along the production direction, until the displacement increased to 70% of the sample height. Samples were placed at the center of the circular plate and only the upper plate was allowed to move downwards to compress the samples, and the lateral expansion was not restricted, as illustrated in Figure 3a. The force and displacement were recorded and the stress–strain curves could be obtained. The deformation of the samples was captured through a digital camera. As shown in the Figure 3b, the elastic modulus (*E*) was determined by the slope of the straight line within the elastic region of the stress–strain curves, the yield strength (*σ_s_*) was determined by the intersection of the stress–strain curve at a 0.2% offset line parallel to the elastic line, and the compressive strength (*σ_c_*) was obtained through the first peak stress in the curve.

### 2.5. CFD Analysis

CFD simulations were carried out to examine the dependency of the permeability and velocity distributions of the optimized unit cell. The pressure drop and permeability were used to quantify transmission performance between different structures. The boundary conditions for the CFD analysis are shown in Figure 3c. Due to the repeatability and the symmetry of the scaffold, the number of units in the three orthogonal directions were constructed as 1 × 1 × 4 to reduce the simulation time. The fluid domains above the scaffold were added to avoid the boundary effect. The flow direction of the fluid at the inlet was vertical, and the velocity was 1 mm/s. The pressure of the outlet was set to 0 Pa and a no-slip condition was applied on the walls. For the analysis, water was selected as the fluid with a density of 1 g/mm^3^ and a viscosity of 1 × 10^−9^ MPa·s. In this work, the simulation was conducted obeying the Navier–Stokes equation and then the permeability of each model was calculated according to Equation (2). All the symbols in the equation are listed in Table 2.

(2) $$K=\frac{v\cdot \mu \cdot L}{\Delta P}$$

## 3. Results

### 3.1. Manufacturing Fidelity and Morphological Analysis

**Figure 4:**
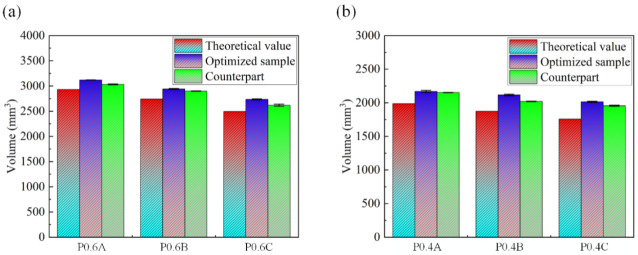
Comparison between the theoretical volume and actual volume of (**a**) P0.6 and (**b**) P0.4.

The volume between the fabricated samples and the idealized CAD models are compared and presented in Table 3 and Figure 4. The obtained results indicate an increase in the volume compared to the design models with a deviation from design value in the range of 3.39–15.12%, and the volume deviation of the optimized samples is larger than the same volume samples.

**Figure 5:**
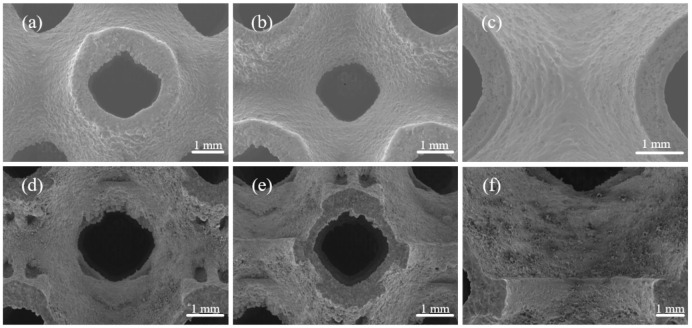
SEM images of the surface of fabricated parts. (**a**–**c**) Outer surface of P0.6C1 and (**d**–**f**) inner surface of P0.6C1.

Figure 5 shows the SEM images of the outer surface and inner surface of an optimized P-TPMS scaffold. In general, no macro-defects (cracks, pores, and deformation) are observed, showing good printability of the samples. The staircase effect can be seen along the building direction and some particles are adhered to the surface. Additionally, it can be noted that excess powders are adhered to the surface of the sample, especially for the downward-facing surface and lateral surface, resulting in the increased thicknesses. As for the bottom surface, a notably rough morphology can be observed due to a large amount of released heat during the solidification of the printed layers, resulting in the adjacent powder melting and sticking to the surface [21]. As for the lateral surface, there are metal powders hanging on the surface because there is no support at the sharp corners of the edges during the manufacturing process. When comparing the inner and outer surfaces, it can be found that the inner surface is rougher due to the topology optimization. The reason behind this is that the sheet thickness of the optimized samples is constantly changing compared to the samples with a uniform sheet thickness, while the diameter of the laser beam is constant during the layer-by-layer processing process.

**Figure 6:**
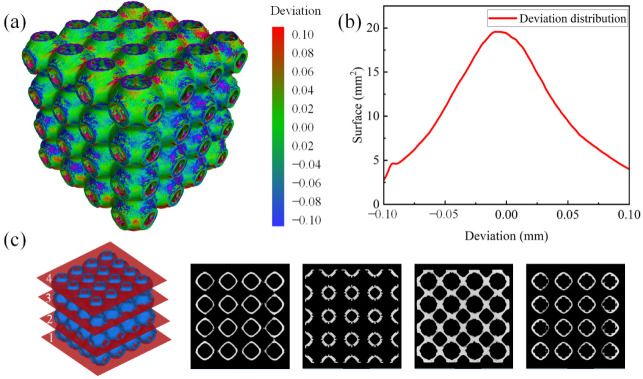
The evaluation of the fabricated samples of P0.6C1 with micro-CT images. (**a**) The 3D deviation maps; (**b**) distribution of deviations over the surfaces; (**c**) tomographic slices of the CT models.

The manufacturing accuracy of the as-built samples is further analyzed using CT scans. Figure 6a,b shows the deviation maps of the reconstruction models and the deviation distribution of the optimized scaffolds. It is indicated that the main deviations distribute in the range from −0.1 mm to 0.1 mm, and the majority of the deviation is around 0 mm, which demonstrates that the deviation can be controlled at a very limited level. Furthermore, Figure 6c shows micro-voids and cracks. These internal voids are inevitable for AM parts and generally attributed to the trapped gas and insufficient fusion of the powder during the manufacturing process [34]. Overall, the printed samples are consistent with the CAD models, there are no obvious defects or damaged cells on the printed samples and the deviation can be controlled effectively during the AM process.

### 3.2. Mechanical Properties

**Figure 7:**
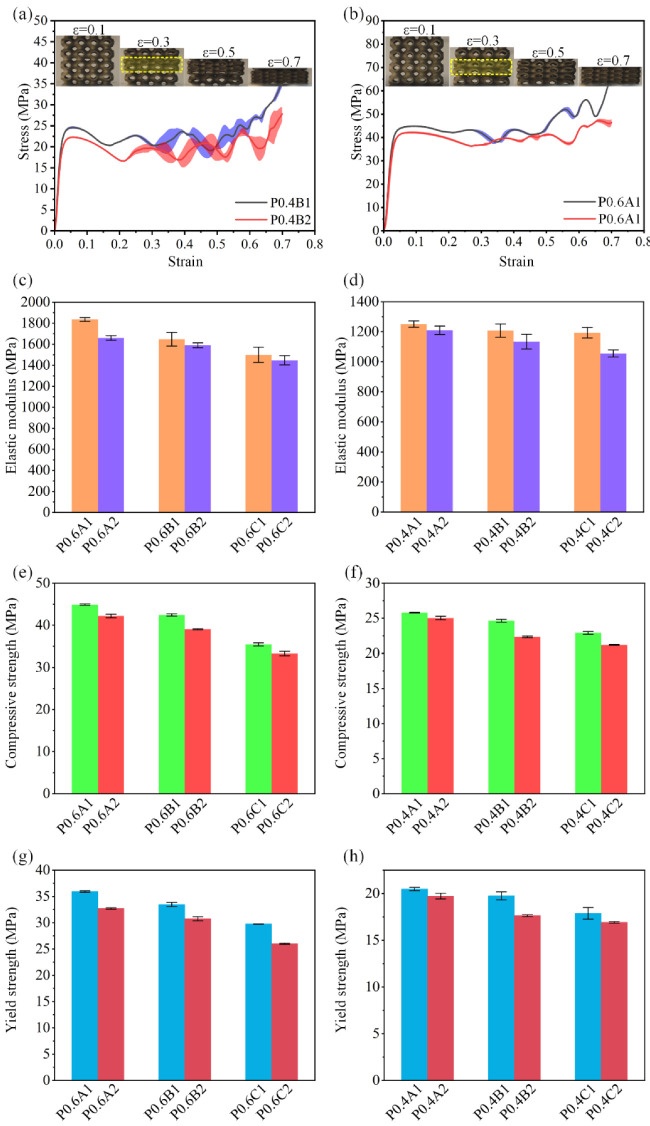
Results of compression tests. (**a**,**b**) Stress–strain curves of P0.4B1 and P0.6A1. (**c**–**h**) Comparison of elastic modulus, compressive strength, and yield strength.

Figure 7a,b shows the compressive stress–strain curves and the captured images of the compressive deformations and failure mechanisms of P0.4A and P0.6C at four different strain levels (10%, 30%, 50%, and 70%). It is found that all samples are quite similar in the compressive deformations and failure mechanisms. The failure starts from the middle layers, showing a horizontal and progressive collapse layer by layer. As for the stress–strain curves, four stages can be observed according to the trend of the curves. Specifically, the curves initially rise up with a constant slope, which is called the elastic stage. The second stage starts when the curves depart from the linear response and entered the non-linear regime, where stress keeps increasing and reaches an initial peak at approximately 0.04–0.06 strain. After the peak stress, the sample begins to show obvious deformation, followed by drop in the curves. Then, it enters the third stage of plateau stress, where the deformation keeps taking place, and some noticeable stress fluctuations in the curve with the continuous increase in strain are observed. Compared to scaffolds with 0.6 mm thickness, the scaffolds with 0.4 mm thickness experience severe fluctuations, which are mainly due to the stress softening caused by the buckling of the local unit cell [22]. The plateau stage continued until the onset of the densification at around 0.6–0.7 strain, then the whole scaffold failed and the curve started to raise dramatically.

The mechanical properties of all samples including elastic modulus, compressive strength, and yield strength are illustrated in Figure 7c–h. The mechanical properties of all samples are improved with the decrease in the porosities, and this is the same trend as the experimental results of other scholars [14,21,35]. The Gibson–Ashby model is the most notable and commonly accepted model for the prediction of the mechanical performance of porous scaffolds. This model indicated that the mechanical performance of porous scaffolds is positively correlated with the porosity, or relative density [36]. It can be observed that the elastic modulus, compressive strength, and yield strength of all scaffolds increases with relative density increases, which is consistent with expectations of the Gibson–Ashby model. However, there is no linear relationship between the optimization degree of the scaffolds and the performance improvement. Figure 7c,d shows the elastic modulus of the scaffolds; the elastic modulus of P0.4 and P0.6 are improved in range of 3.4–13.1% and 3.5–10.6%, respectively. For P0.4, P0.4B has the largest optimization degree:, as the elastic modulus increases from 1056.6 MPa to 1193.9 MPa by 13.1%. For P0.6, P0.6A has the largest optimization degree, as the elastic modulus increase from 1660.0 MPa to 1836.0 MPa by 10.6%, but the P0.6B and P0.6C scaffolds have a relatively low improvement, which is only 3.5%.

Moreover, it can be found from Figure 7e,f that the compression strength of all P0.6 scaffolds were improved to roughly the same degree in the range of 6.4–8.7%. However, for the compression strength of P0.4 scaffolds, the improvement of P0.4A was only 3.4%, while the improvement of the P0.4B and P0.4C structures reached a relatively high level of 10.2% and 8.2%, respectively. In addition, we can conclude from Figure 7g,h that the increase in yield strength of P0.6 scaffolds was generally at a relatively high level, with the minimal degree of 8.8%. For the P0.4 scaffolds, only P0.4B had a 12.0% increase, while the increases in P0.4A and P0.4C were only 3.8% and 5.7%, respectively.

### 3.3. Fluid Permeability

**Figure 8:**
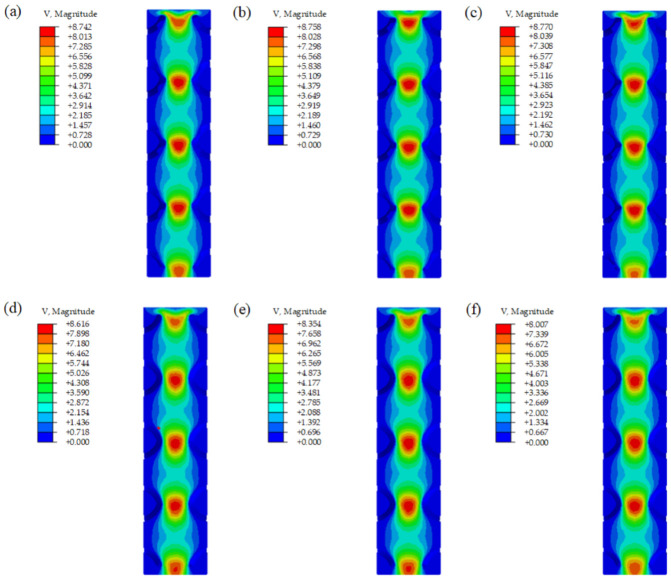
Velocity contours from CFD analysis of (**a**) P0.6A1, (**b**) P0.6B1, (**c**) P0.6C1, (**d**) P0.6A2, (**e**) P0.6B2, and (**f**) P0.6C2.

**Figure 9:**
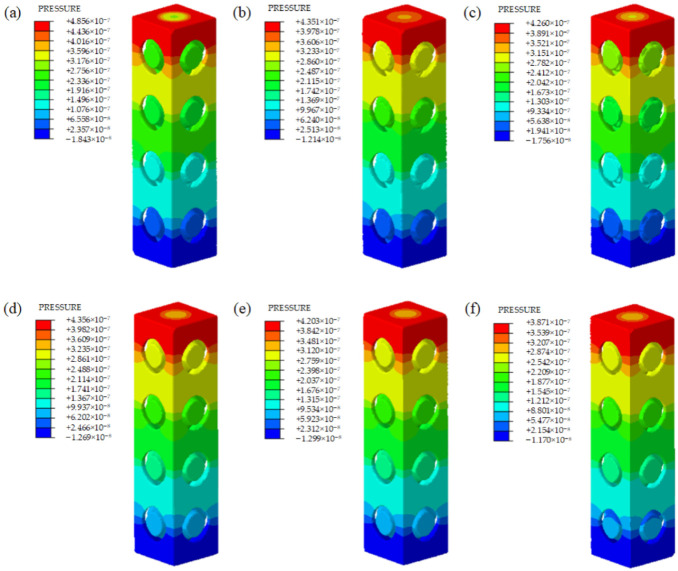
Pressure contours from CFD analysis of (**a**) P0.6A1, (**b**) P0.6B1, (**c**) P0.6C1, (**d**) P0.6A2, (**e**) P0.6B2, and (**f**) P0.6C2.

**Figure 10:**
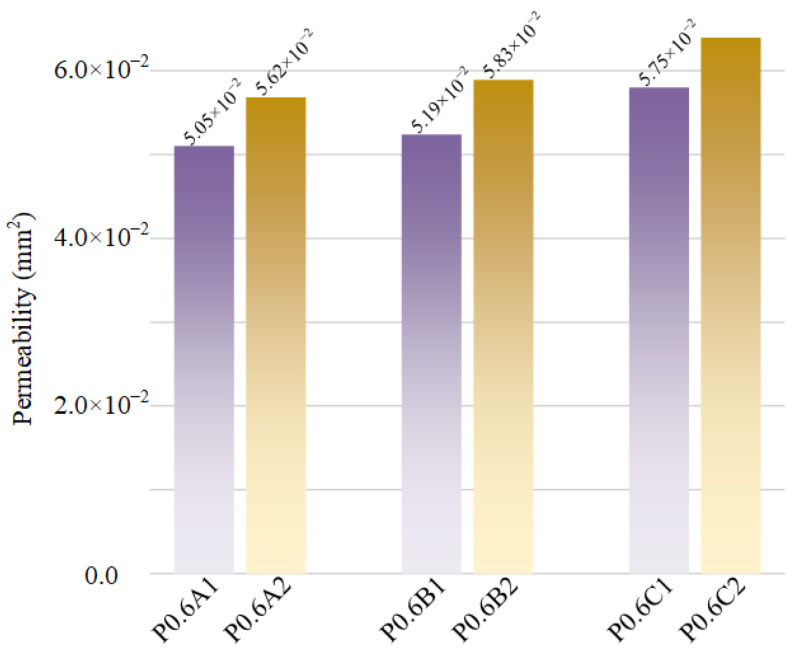
Comparison of the calculated permeability of different P0.6 scaffolds.

Figure 8 shows the velocity distributions of several representative samples from the CFD simulation. To compare the mass transport properties among different scaffolds, the pressure drop (Δ*P*) and the permeability (*K*) values of each model were calculated. Figure 9 and Figure 10 show the pressure drop and the permeability of all scaffolds with 0.6 mm sheet thickness.

Similar velocity distribution can be observed for both scaffolds with either uniform sheet thickness or optimized sheet thickness. The velocity distribution is irregular, except in the vertical direction. The velocity contours show that the flow velocity at the center of the entrance and outlet of the unit cell is the highest, and gradually decreases along the radial direction from the center to the internal boundary. The speed in the center of the unit structure is high, which is beneficial for transporting the cells and nutrients and oxygen through the whole scaffold. The lower speed can be seen around areas that are closer to the boundary of the internal curved surface. This phenomenon is conducive to the adsorption of cells and nutrients on the inner surface of the scaffold. The flow rate of the optimized unit cells is higher than the same volume unit cells, and it increases slowly and basically remaines at a fixed value with the improvement of the optimization degree of unit cells, but the flow rate of the unit cell with uniform sheet thickness decreases gradually. This is because the sheet thickness of the same volume unit cell gradually decreases and larger inlet and outlet pores can be observed. These results indicate that the degree of optimization barely affects the flow rate, and the main factor affecting the flow rate is the size of the inlet and outlet pores of the unit. We can also observe that the high-velocity area of the same volume unit cells is more elongated, while the high-velocity area of optimized unit cells is shorter. This phenomenon is caused by the change in the inner wall of the optimized unit cells.

As shown in Figure 9, the pressure distribution is uniform between different structures and the pressure has a linearly gradient decreasing tendency from the inlet to the outlet among all scaffolds. According to the permeability results in Figure 10, the unit cells with a higher optimization degree have higher permeability, and the optimized unit cells have higher permeability than the scaffolds with uniform sheet thickness. The reason is that more materials of unit cells were removed with the topology optimization, and the unit cells gained larger space inside, and in general, higher porosity results in higher permeability. After topology optimization, many potholes appeared on the inner surface of the unit cells, which makes the internal structure more complex and causes the surface area and surface frictional forces to increase, which is more conducive to cell adsorption. The permeability of this study ranges from 50.5 × 10^−9^ m^2^ to 63.3 × 10^−9^ m^2^, and the values are in the range of previous results on porous scaffolds, and they are slightly larger than that of some human bones [30]. However, they are of the same order of magnitude, and the permeability of trabecular bones is dependent on the region of the human body, and the span of the permeability of human bones is large [15]. However, compared with CT-reconstructed model, the idealized CAD model has certain limitations in the CFD simulations. This is because the as-built samples have some dimension deviations with idealized CAD models. Compared with the idealized CAD model, the as-built samples have some dimension deviations, which would affect the mass transport characteristics. To further investigate the permeability of the model, more details on the modeling with further analysis are required in future works.

## 4. Conclusions

This study designed a novel P-TPMS scaffold with an optimized thickness of two different thicknesses and three different optimization degrees. To verify the performance of the proposed P-TPMS scaffolds, the morphology, mechanical properties, and permeability were comprehensively investigated and compared. The main findings and conclusions of this study are as follows:

(1) Combining the micro-CT reconstructed models and captured SEM images, no macro-defects were observed, only some particles adhered to the surface and some microstructural defects in the form of micro-voids and cracks. Samples with optimized sheet thickness had larger volume deviation and a rougher inner surface than the samples with uniform sheet thickness. The majority of the deviations of the optimized scaffolds were around 0 mm. Therefore, the designed scaffolds could be additively fabricated by SLM successfully with acceptable deviations.
(2) In terms of mechanical behavior, the elastic modulus, yield strength, and compressive strength of the samples with optimized sheet thickness were greater than the samples with uniform sheet thickness. However, the degree of optimization of the scaffolds had no linear relationship with the performance improvement. In addition, all samples exhibited similar failure modes. The mechanical properties of all the samples increased with increasing relative density, which is consistent with expectations of the Gibson–Ashby model.
(3) The CFD simulation demonstrated that all of the samples had similar velocity distribution and pressure distribution. The velocity showed a slight upward trend with the improvement of the optimization degree, and the optimized unit cells had higher velocity compared to the same volume unit cells. However, the permeability of scaffolds with optimized sheet thickness was slightly smaller than that of the uniform sheet thickness ones.

In summary, the mechanical properties and permeability of scaffolds designed using the proposed method were optimized to some extent, and the scaffolds were found suitable for use in bone scaffolds. The work in this study provides valuable guidance on designing and manufacturing porous scaffolds. In our future work, we will study the selection criterion for the optimization, as well as the effects of optimization degree on performance improvement, in order to achieve the optimal configurations.

## Figures and Tables

**Table 1:** Chemical composition of the 304L stainless steel powders.

Element	Fe	Si	Cr	Ni	Mn	C	O	S	P
wt%	Bal.	0.43	19.35	9.53	0.71	0.006	0.056	0.005	0.010

**Table 2:** Parameters for calculating the permeability.

Symbol	Description	Unit
*K*	the permeability coefficient	mm^2^
*L*	the length of the model	mm
Δ*P*	pressure difference	MPa
*μ*	the dynamic viscosity coefficient of the fluid	MPa·s
*v*	the velocity of the fluid	mm/s

**Table 3:** Volume, mass, and densities of samples.

Type	Actual Volume (mm^3^)	Theoretical Volume (mm^3^)	Mass (g)	Average Relative Density (%)	Deviation (%)
P0.4A1	2169.62 ± 15.29	1988.59	17.14 ± 0.12	14.71 ± 0.10	9.45 ± 0.77
P0.4A2	2153.59 ± 1.58	1988.59	17.01 ± 0.01	14.60 ± 0.01	7.95 ± 0.08
P0.4B1	2117.72 ± 12.70	1875.28	16.73 ± 0.10	14.36 ± 0.09	13.45 ± 0.68
P0.4B2	2021.94 ± 4.18	1875.28	15.97 ± 0.03	13.71 ± 0.03	7.33 ± 0.22
P0.4C1	2015.19 ± 9.47	1760.67	15.92 ± 0.07	13.66 ± 0.06	15.12 ± 0.54
P0.4C2	1956.96 ± 7.80	1760.67	15.46 ± 0.06	13.27 ± 0.05	10.51 ± 0.44
P0.6A1	3118.57 ± 2.15	2933.09	24.64 ± 0.02	21.14 ± 0.01	6.32 ± 0.07
P0.6A2	3032.49 ± 7.33	2933.09	23.96 ± 0.06	20.56 ± 0.05	3.39 ± 0.25
P0.6B1	2940.93 ± 10.20	2744.79	23.23 ± 0.08	19.94 ± 0.07	7.15 ± 0.37
P0.6B2	2899.58 ± 4.18	2744.79	22.91 ± 0.03	19.66 ± 0.03	5.64 ± 0.15
P0.6C1	2735.44 ± 8.20	2493.18	21.61 ± 0.06	18.55 ± 0.06	9.72 ± 0.33
P0.6C2	2618.99 ± 19.64	2493.18	20.69 ± 0.16	17.76 ± 0.13	5.05 ± 0.79

## References

[1] Yang L., Yan C., Fan H., Li Z., Cai C., Chen P., Shi Y., Yang S. (2019). Investigation on the Orientation Dependence of Elastic Response in Gyroid Cellular Structures. J. Mech. Behav. Biomed. Mater..

[2] Kader M.A., Hazell P.J., Brown A.D., Tahtali M., Ahmed S., Escobedo J.P., Saadatfar M. (2020). Novel Design of Closed-Cell Foam Structures for Property Enhancement. Addit. Manuf..

[3] Maggi A., Li H., Greer J.R. (2017). Three-Dimensional Nano-Architected Scaffolds with Tunable Stiffness for Efficient Bone Tissue Growth. Acta Biomater..

[4] Zhang L., Song B., Yang L., Shi Y. (2020). Tailored Mechanical Response and Mass Transport Characteristic of Selective Laser Melted Porous Metallic Biomaterials for Bone Scaffolds. Acta Biomater..

[5] Diloksumpan P., De Ruijter M., Castilho M., Gbureck U., Vermonden T., Van Weeren P.R., Malda J., Levato R. (2020). Combining Multi-Scale 3D Printing Technologies to Engineer Reinforced Hydrogel-Ceramic Interfaces. Biofabrication.

[6] Paré A., Charbonnier B., Tournier P., Vignes C., Veziers J., Lesoeur J., Laure B., Bertin H., De Pinieux G., Cherrier G. (2020). Tailored Three-Dimensionally Printed Triply Periodic Calcium Phosphate Implants: A Preclinical Study for Craniofacial Bone Repair. ACS Biomater. Sci. Eng..

[7] Parthasarathy J., Starly B., Raman S. (2011). A Design for the Additive Manufacture of Functionally Graded Porous Structures with Tailored Mechanical Properties for Biomedical Applications. J. Manuf. Process..

[8] Yang N., Gao L., Zhou K. (2015). Simple Method to Generate and Fabricate Stochastic Porous Scaffolds. Mater. Sci. Eng. C.

[9] Kadkhodapour J., Montazerian H., Darabi A.C., Zargarian A., Schmauder S. (2017). The Relationships between Deformation Mechanisms and Mechanical Properties of Additively Manufactured Porous Biomaterials. J. Mech. Behav. Biomed. Mater..

[10] Cheng X.Y., Li S.J., Murr L.E., Zhang Z.B., Hao Y.L., Yang R., Medina F., Wicker R.B. (2012). Compression Deformation Behavior of Ti-6Al-4V Alloy with Cellular Structures Fabricated by Electron Beam Melting. J. Mech. Behav. Biomed. Mater..

[11] Hollister S.J. (2005). Porous Scaffold Design for Tissue Engineering. Nat. Mater..

[12] Bose S., Vahabzadeh S., Bandyopadhyay A. (2013). Bone Tissue Engineering Using 3D Printing. Mater. Today.

[13] Davoodi E., Montazerian H., Mirhakimi A.S., Zhianmanesh M., Ibhadode O., Shahabad S.I., Esmaeilizadeh R., Sarikhani E., Toorandaz S., Sarabi S.A. (2022). Additively Manufactured Metallic Biomaterials.

[14] Ma S., Tang Q., Han X., Feng Q., Song J., Setchi R., Liu Y., Liu Y., Goulas A., Engstrøm D.S. (2020). Manufacturability, Mechanical Properties, Mass-Transport Properties and Biocompatibility of Triply Periodic Minimal Surface (TPMS) Porous Scaffolds Fabricated by Selective Laser Melting. Mater. Des..

[15] Bobbert F.S.L., Lietaert K., Eftekhari A.A., Pouran B., Ahmadi S.M., Weinans H., Zadpoor A.A. (2017). Additively Manufactured Metallic Porous Biomaterials Based on Minimal Surfaces: A Unique Combination of Topological, Mechanical, and Mass Transport Properties. Acta Biomater..

[16] Kapfer S.C., Hyde S.T., Mecke K., Arns C.H., Schröder-Turk G.E. (2011). Minimal Surface Scaffold Designs for Tissue Engineering. Biomaterials.

[17] Yang N., Quan Z., Zhang D., Tian Y. (2014). Multi-Morphology Transition Hybridization CAD Design of Minimal Surface Porous Structures for Use in Tissue Engineering. CAD Comput. Aided Des..

[18] Poltue T., Karuna C., Khrueaduangkham S., Seehanam S., Promoppatum P. (2021). Design Exploration of 3D-Printed Triply Periodic Minimal Surface Scaffolds for Bone Implants. Int. J. Mech. Sci..

[19] Blanquer S.B.G., Werner M., Hannula M., Sharifi S., Lajoinie G.P.R., Eglin D., Hyttinen J., Poot A.A., Grijpma D.W. (2017). Surface Curvature in Triply-Periodic Minimal Surface Architectures as a Distinct Design Parameter in Preparing Advanced Tissue Engineering Scaffolds. Biofabrication.

[20] Wang S., Shi Z., Liu L., Zhou X., Zhu L., Hao Y. (2020). The Design of Ti6Al4V Primitive Surface Structure with Symmetrical Gradient of Pore Size in Biomimetic Bone Scaffold. Mater. Des..

[21] Al-Ketan O., Rowshan R., Abu Al-Rub R.K. (2018). Topology-Mechanical Property Relationship of 3D Printed Strut, Skeletal, and Sheet Based Periodic Metallic Cellular Materials. Addit. Manuf..

[22] Sun Q., Sun J., Guo K., Wang L. (2022). Compressive Mechanical Properties and Energy Absorption Characteristics of SLM Fabricated Ti6Al4V Triply Periodic Minimal Surface Cellular Structures. Mech. Mater..

[23] Novak N., Al-Ketan O., Krstulović-Opara L., Rowshan R., Abu Al-Rub R.K., Vesenjak M., Ren Z. (2021). Quasi-Static and Dynamic Compressive Behaviour of Sheet TPMS Cellular Structures. Compos. Struct..

[24] Li X., Xiao L., Song W. (2021). Compressive Behavior of Selective Laser Melting Printed Gyroid Structures under Dynamic Loading. Addit. Manuf..

[25] Li J., Chen D., Fan Y. (2019). Evaluation and Prediction of Mass Transport Properties for Porous Implant with Different Unit Cells: A Numerical Study. Biomed Res. Int..

[26] Santos J., Pires T., Gouveia B.P., Castro A.P.G., Fernandes P.R. (2020). On the Permeability of TPMS Scaffolds. J. Mech. Behav. Biomed. Mater..

[27] Castro A.P.G., Pires T., Santos J.E., Gouveia B.P., Fernandes P.R. (2019). Permeability versus Design in TPMS Scaffolds. Materials.

[28] Gómez S., Vlad M.D., López J., Fernández E. (2016). Design and Properties of 3D Scaffolds for Bone Tissue Engineering. Acta Biomater..

[29] Ali D., Ozalp M., Blanquer S.B.G., Onel S. (2020). Permeability and Fluid Flow-Induced Wall Shear Stress in Bone Scaffolds with TPMS and Lattice Architectures: A CFD Analysis. Eur. J. Mech. B/Fluids.

[30] Asbai-ghoudan R., Ruiz S.G., Rodriguez-florez N. (2021). Analytical Model for the Prediction of Permeability of Triply Periodic Minimal Surfaces. J. Mech. Behav. Biomed. Mater..

[31] Jia H., Lei H., Wang P., Meng J., Li C., Zhou H., Zhang X., Fang D. (2020). An Experimental and Numerical Investigation of Compressive Response of Designed Schwarz Primitive Triply Periodic Minimal Surface with Non-Uniform Shell Thickness. Extrem. Mech. Lett..

[32] Wang H., Tan D., Liu Z., Yin H., Wen G. (2022). On Crashworthiness of Novel Porous Structure Based on Composite TPMS Structures. Eng. Struct..

[33] Liu B., Liu M., Cheng H., Cao W., Lu P. (2022). A New Stress-Driven Composite Porous Structure Design Method Based on Triply Periodic Minimal Surfaces. Thin-Walled Struct..

[34] Jin Y., Zou S., Pan B., Li G., Shao L., Du J. (2022). Biomechanical Properties of Cylindrical and Twisted Triply Periodic Minimal Surface Scaffolds Fabricated by Laser Powder Bed Fusion. Addit. Manuf..

[35] AlMahri S., Santiago R., Lee D.W., Ramos H., Alabdouli H., Alteneiji M., Guan Z., Cantwell W., Alves M. (2021). Evaluation of the Dynamic Response of Triply Periodic Minimal Surfaces Subjected to High Strain-Rate Compression. Addit. Manuf..

[36] Maconachie T., Leary M., Lozanovski B., Zhang X., Qian M., Faruque O., Brandt M. (2019). SLM Lattice Structures: Properties, Performance, Applications and Challenges. Mater. Des..

